# Thoughts on the Tongue as an Element in Diagnosis

**Published:** 1846-10

**Authors:** John Dawson

**Affiliations:** Jamestown, Ohio


### WESTERN JOURNAL

OF

### MEDICINE AND SURGERY.

OCTOBER, 1846.

---

### Art. I.—*Thoughts on the Tongue as an element in Diagnosis.* By John Dawson, M.D., of Jamestown, Ohio.

Our intention in this paper is to offer some thoughts on the *Tongue as an Element of Diagnosis.* For centuries the appearances of this organ have been consulted as being of great utility in indicating the character of diseases general and local. The ancient authors, to whom we have had access, have left sufficient upon record to show the high estimation in which its different appearances were regarded by them. By the schools of Abernethy and Broussais the value of these morbid appearances have, perhaps, been overrated. Both of these eminent men, and their followers, regard the tongue as reflecting the exact morbid condition of the alimentary canal. As we shall subsequently see, this position will have to be classed among the things that are obsolete. It should not, however, be supposed from this that the appearances of the tongue are not among the most available means we possess of arriving at a correct knowledge of many pathological states of the system. There are some things that may be known by their inspection, and there are other things that require the consultation of all the phenomena presented; and even then, much as the scope has been enlarged by the labor of modern times, it is still, in many instances, too limited for accurate diagnosis.

We may know, as a general rule, that the system is in a state of disease by the appearance of the tongue, but the *kind* and *character* of the disease can often only be ascertained by reference to other and more available signs.

In order to present some views on the subject before us, we will in the first place give the result of our own observations on the changes which the tongue has undergone in some of the diseases of the general system which we have been called upon to treat; also on the changes it has undergone in diseases of particular organs, tissues, &c.; and in the last place, offer some suggestions on the import of these several morbid appearances.

Among the general diseases giving rise to modifications in the appearance of the tongue, because of the frequency of its occurrence, we have paid the most attention to fever. All varieties of what are generally termed idiopathic fever have exhibited morbid appearances in greater or less perfection.

In the *synochus* fever of British and Continental writers, usually in this country termed *remittent*, the tongue has pretty constantly been more or less altered in appearance. Altogether the most constant change that we have observed has been in relation to its surface. A great proportion of the cases have shown this to be coated *white* at the beginning of the malady. After continuing for a while the coat has amounted to what is generally termed load. At the commencement the coat has usually been so light as not to cover up entirely the papillæ; and, as a consequence from this circumstance, not less, perhaps, than from a hypertrophied condition, the papillæ have commonly exhibited a protruded appearance. Sometimes this has continued during the whole course of the complaint; but oftener, by the upper surface becoming loaded, the papillæ have been concealed. By a number of circumstances the white color has been varied. The continuance of the disorder for any length of time, by which the secretions from the mouth had become vitiated, have given rise to a dirty, and in some instances, a brownish appearance of the coat. When, also, at any period during the course the liver has been implicated it has happened that the coat or load partook more or less of the icteroid hue common to other parts of the system. This, however, has only been occasionally the case in what is called epidemic bilious fever; for I have witnessed a majority of cases of bilious fever during the past autumn when the complaint was epidemic, in which the diffusion of bilious matter through the system, so as to color the tongue, skin, and eyes, was not present. To some extent, we think, the coat of the tongue has been varied in color by the nature of the remote cause. A few cases were observed that from the beginning were characterized with a high grade of vascular excitement, urgent thirst, a hot and dry skin. In such the tongue, although very white at first, very soon became brown and dry, and in some instances black. Besides, the changes of color referable to the coat, the proper substance of the organ itself underwent some important modifications. In these cases characterized by strong febrile excitement, the tongue was pretty constantly red on its edges, under surface, and apex. Milder grades of excitement were attended with a pale, in some instances, a livid appearance, which continued during the whole course, unless inflammation of some important organ supervened. Great redness of the tongue at the commencement of the disease was usually found associated with a vigorous, robust system, and much functional energy; while the pale appearance was found in connexion with an effeminate condition of the general system or broken down constitution. The fever generally denominated remittent has prevailed in this locality a few years past, and almost every case of which was attended with but a very mild grade of febrile excitement, scarcely any thirst, and not much heat or dryness of the skin. In such cases the tongue, at first, was nearly normal, perhaps paler than in health, and often continued in this condition during the course. Some of these cases were very protracted, and in the latter stages gave evidence of disease of the Peyerian glands. When this happened the tongue became redder, more granulated, and exhibited a tendency to dryness. The condition of the tongue in the complaint before us, so far as dryness or moisture was concerned, depended upon several circumstances. As soon as the malady was completely developed patients generally complained more or less of dryness in the mouth, tongue, and fauces. On inspection, nothing in the majority of cases could be observed adequate to account for such a sensation. From the beginning there were cases, nevertheless, in which there was a predisposition to visible dryness. We witnessed an example of this kind in a man aged about 40 years. He was taken in autumn with bilious fever, and was sick a few days before we saw him. On the first inspection his tongue was observed to be remarkably dry, somewhat chapped, and the coat on it had rather the color of mahogany. We looked upon this appearance in connexion with other phenomena present, as ominous of a fatal issue, and such proved to be the result. He was put on mercurials and other means addressed to what was supposed to be gastro-intestinal irritation of a high order, but without any success, for the dryness of the tongue and inside of the mouth increased, the brain became implicated, and he expired. An intelligent practitioner, a short time since, related to me a case supposed to be malignant intermittent, or what is more properly termed congestive fever, in which the tongue was remarkably dry. This case was put on mercurials, and under their use the tongue became moist and assumed a very favorable appearance. The patient, nevertheless, died in a state of profound coma shortly after the favorable alteration occurred.

We witnessed the case of a lady of middle age taken with the premonitory symptoms of bilious fever. On the third or fourth day of her illness we saw her for the first time. On its being examined, the tongue, at this period, was found to be extremely red, perfectly clean, as smooth on its upper surface as if it had been varnished, with considerable tendency to become dry. Associated with these phenomena there were tenderness in the epigastrium, thirst, and dysenteric discharges from the bowels. The tendency to dryness in this case continued for about twenty days, though the tongue at no time became chapped. The patient recovered under the use of medicines prescribed for an irritated condition of the bowels.

Alterations relating to the form of the tongue have so seldom been well defined in the complaint of which we have been speaking, that we can say but little concerning them. We are inclined to think that the organ has seldom, if ever been swollen, but in a few cases, on the contrary, rather contracted and pointed. It is true that we have seen a number of cases in which the organ appeared flabby and pale, but upon inquiry, such appearances could always be traced to some fault in the constitution—dyspepsia, or to something else having a tendency to impair the nutritive function.

Enlarged and elongated papillæ have been observed very frequently, at the commencement of the disease, protruding through the coat and imparting to the tongue the appearance elementary writers usually assign to it in scarlatina. As the disease progressed the papillæ on the surface became covered up by the coat, but those on the apex and edges continued to enlarge, giving rise to a granulated appearance. In pretty much all the cases characterized by this modification of the papillæ there was a great tendency to prostration. When not subdued very early, the affection became complicated with diarrhœa, epigastric tenderness, in some instances, meteorism, coma, and other typhoid symptoms.

The motions of the tongue have not been so strikingly characterized in this as in cases we have witnessed of typhus. When dry and chapped, patients have complained of being unable to control its motions; and when requested to protrude it have expressed their inability to do so. Very much debilitated with the continuance of the disease upon them for a considerable length of time, patients have complained of inability to protrude the tongue, and when insisted upon to pass it over the teeth for inspection, the organ appeared tremulous. We have noticed a few cases of this kind in which the organ from lying against the teeth had become indented. We will now give a sketch of our observations of the

*Condition of the tongue in epidemic typhus fever.*—This fever has prevailed several times in this region as an epidemic. In most of the cases, the tongue, during the period of incubation, which generally lasted from one to two weeks, presented rather an exsanguineous appearance, was slightly coated, and attended with no perceivable change of form. As the disease progressed it was usual for the color to change to a brighter red, for the coat to increase in thickness, and also for the coat to become discolored, becoming brown in some cases, in others almost black. As a general rule the papillæ were more or less enlarged, protruding through the coat when it was thin, and giving rise to a granulated appearance on the edges and apex. When the complaint was of considerable duration, the coat increased in thickness to the degree generally entitled load. This in some instances continued during the whole period of the complaint; in other instances, by the epithelium becoming detached, it sloughed off in flakes leaving the surface under it red and very tender. The tendency to dryness was exhibited in but very few cases; and invariably these were characterized by evidence of a high degree of gastric and intestinal irritation. Commonly the motions of the tongue were unimpaired. In a few cases, very much debilitated by the great duration of the malady, patients were inclined to speak only a word or two at a time, and with long intervals between them; and when asked to protrude the tongue, they would express an inability to do so. On witnessing two or three epidemics of typhus, we saw no case, in which from the commencement, the tongue was black. This phenomenon occurred only in the advanced stages and like persistent dryness, it was generally followed by a fatal issue.

The condition of the tongue in diseases strictly inflammatory has of late been made the subject of extensive clinical research. This class, comprehensive enough in the estimation of some to supersede the necessity of all other branches of nomenclature, is characterized with such a diversity in the character of the symptoms generally, and also in the morbid appearances of the tongue, that it is very difficult to give the condition of this organ with any degree of precision.

Inflammation resulting from fractures and extensive injuries of the soft parts, we have constantly observed, have been attended with a white coat. While this phenomenon has universally been present, the appearances in other respects have varied according to the intensity of the affection, nature of the organ or tissue involved, diathesis of the patient, &c.

Injuries of the osseous tissues and soft parts contiguous, when extensive, have, without a single exception, been immediately followed with a coated tongue, the coat always very white at first. Attended with a high fever, the tongue in some instances has been disposed to dryness, and in cases of slow recovery, to a red or granulated state of its edges.

Acute inflammation of any of the more important organs of the body, as far as we have observed, has been connected with a morbid state of the tongue, which varied according to circumstances. As a general rule, during the first stages, the tongue was coated; after this period, if the complaint proved stubborn, other changes occurred, such as the upper surface becoming loaded, brown, or perhaps dry and chapped.

We observed in a case of inflammation of the mucous surface of the bowels, proceeding as we supposed from malaria, that the tongue after being coated turned brown and ultimately became dry and almost black. We have also witnessed an acute inflammation of the mucous surface of the stomach associated with delirium tremens, that had associated with it dryness of the tongue and entire inside of the mouth. Both of these cases terminated fatally.

In subacute or chronic inflammation, the tongue, as in the acute form of the disease, had been generally coated, sometimes pale, and we have noticed a few cases in which it was flabby during the whole course of the complaint. Protrusion of the papillæ has been of no unfrequent occurrence here; and we have seen some cases in which the organ was fissured and lobulated.

To a considerable extent the tongue has been varied by the duration of the inflammatory attack. In cases where the affection ran its course rapidly the tongue has exhibited a tendency to become dry and chapped. On the contrary, in cases of a protracted character, it has been more apt to become loaded, foul, and more or less discolored. By the nature of the tissue or organ affected with inflammatory action, the appearances have been modified. In glossitis we are told by elementary writers that the tongue soon becomes red, hot, and dry. Its form is almost immediately altered, being at times so much hypertrophied as to fill up the entire cavity of the mouth.

As we witnessed a case of glossitis in connexion with epidemic erysipelas that was strongly characterized, we will transcribe a brief account of it from the Western Journal of Medicine and Surgery in which it was published in August last. The subject of it was a man of strong constitution, plethoric habit, and 46 years of age. On the 4th of May last he was taken with the symptoms of epidemic erysipelas; on the 8th, for the first time, he commenced complaining of his tonge. It presented on examination at this period a healthy appearance, except that it was slightly coated and the papillæ somewhat enlarged. On the 9th it was painful, and considerably enlarged, though not altered in color. At this time the secretions from it and the salivary glands, were abundant. It continued to enlarge on the 10th, and on the 11th it had attained a size sufficient to fill the entire cavity of the mouth, and protruded for some distance between the teeth, from which circumstance the patient's jaws were forced apart, and he became unable to articulate. At this period its color was redder than formerly, though it still continued moist, and its upper surface was very heavily loaded. It had been scarified on the 9th, and from these places there issued a thick dense layer of coagulable lymph or false membrane. On the 13th, the swelling began to subside, and the surface to clean off. Although the complaint with which this condition of the tongue was connected, was provincially termed black-tongue we saw but this one instance in which the organ was prominently affected. Nor have our researches, concerning the observations of others, justified those revolting rumors about the prevalence of black-tongue with which our country twelve months ago was so rife. The tongue in the complaint of which we have been speaking was usually white at the commencement, becoming, as the complaint progressed, loaded, foul, or discolored, according to the severity, duration, &c.; and in one case that we attended it was found to be dry on the first day of the disease.

Inflammation of the dermoid tissue, such for example as obtains in exanthematous affections, has presented some peculiarities that deserve a passing notice. In scarlatina the tongue has been redder than in most other complaints, generally coated, and in perhaps one-half of the cases which we have seen there has been an elongation of the papillæ, and a protrusion of them through the coat. This latter condition, we are aware, has been given by most elementary writers as a diagnostic of scarlatina. In this respect, however, it is entitled to no confidence; for it is a very common appearance in children, it matters not with what disease they are affected; and it is also present in a number of diseases to which adults are subject besides scarlatina. Last season we witnessed a case of scarlatina in which the tongue was perfectly clean, of a fiery red color, and as smooth as if it had been glazed, with some tendency to dryness. At the commencement, this condition was present, and continued until convalescence, without being associated with any lesion more than what is common to scarlatina, except a slight diarrhœa. The tongue in rubeola has exhibited nothing interesting or peculiar. Generally it was furred at first, followed by other appearances corresponding to the intensity, duration, &c., of the complaint.

The serous and mucous membranes, when the seat of inflammation, have been attended with nothing entitled to the consideration of a characteristic. A coated tongue has usually been present in both classes of disease, followed by other phenomena corresponding to the grade of excitement, form of the complaint, or idiosyncrasy of the patient. In cases of acute inflammation of the meninges, of the peritoneum, or of the pleura, the tongue has been disposed to become loaded, or dry. The same has been true of the state of this organ in acute inflammation of the mucous membranes. In chronic inflammation of the alimentary canal giving rise to diarrhœa, dyspepsia, &c., the tongue has been often pale, flabby, sometimes broader than natural, and always more or less coated. About the same condition has attended it in the same form of disease in the serous membranes.

*Chlorosis*, as far as we have observed it, has been attended with certain modifications of the tongue that are, perhaps, characteristic. Of the pallid appearance of the general complexion it has very intimately partaken. Always indeed on witnessing the exsanguineous state of the countenance, the same was observable on the tongue. Not constantly, but pretty frequently, in addition to the paleness there have also been tumefactions, occasionally fissures; once in a while the organ has been indented. It has not been common to see it much loaded, still it has been usually coated. In many cases the papillæ have been almost entirely obliterated, giving to the organ an unnaturally smooth appearance.

In diseases of the nervous system the tongue has varied but little from what we have described as being its condition in chlorosis. Perhaps the only real difference was that the phenomena in the latter disease were more permanently and decidedly marked. A thin coat, with paleness, connected at times with a flabby, indented, fissured, or sulcated condition, has been usually present as characteristics of this class of diseases. Persons laboring under what might be termed a nervous diathesis, have had these phenomena most strongly marked.

Having given briefly our experience in some of the more important classes of diseases we will now offer some few thoughts on

*The import of the different appearances of the Tongue.*—Much of the confidence reposed in the different appearances of the tongue, as indicative of certain kinds or grades of diseased action is, by the intelligent part of the profession, about to be suspended. It has been well settled by modern research, that the schools of Broussais and Abernethy are wrong in stating that the tongue will point out the exact state of the alimentary canal. Failing here, where from the similarity of the tissues, and intimate sympathetic relations, it could be most reasonably expected to reflect every change that might occur, of course, its indications concerning diseased actions more remote, and less intimately connected with it, must be regarded as being exceedingly equivocal. It may therefore be taken as a settled position, that, although the state of the tongue is entitled to great consideration as an index to the condition of the general system, yet in order to be entitled to confidence in making out the character or kind of disease, its appearances must coincide with other symptoms.

Before attempting to discuss the import of the different morbid appearances, it will, perhaps, not be out of place to notice the fact that a healthy condition of the organ may exist, and yet, certain parts of the system be the seat of serious disease. The connexion between the state of the tongue, and that of the stomach, has been lately made the subject of extensive clinical observation by Andral and other eminent pathologists. Andral tells us that "we may have a diseased stomach with a healthful condition of the tongue." Speaking of chronic gastritis he says—"The tongue in this affection is far from presenting always the same appearance. First there is a certain number of cases in which it does not at all deviate from its normal state; and what is remarkable this happens precisely in those cases where the stomach has become the seat of the most serious organic alterations. It is principally in fact in cases of cancerous degeneration of the stomach that we have had an opportunity of observing this continuance of the tongue in its normal state." Louis, in giving an account of the gastritis which accompanies fever, states that in many of the worst cases the tongue was natural.

On the contrary, it should be remembered that we may have a diseased condition of the tongue, or rather an appearance of the tongue indicative of disease general, or local, when at the same time the general health is but little or perhaps not at all deranged. In relation to this point, Andral tells us, that "we may have a diseased appearance of the tongue with a healthful state of the stomach." In his last edition of his own and Stokes's Lectures on the Practice of Medicine, Dr. Bell alludes to the case of a person that he knew, who, for twenty-five years never had an entirely clean tongue. On waking every morning this individual would find his tongue dry, fevered, yellow, and often brown; and sometimes giving out a little blood mixed with saliva. Occasionally it resembled that of a patient in the advanced stage of typhoid fever. Yet notwithstanding these appearances the individual "was seldom laid up by sickness." Billings proposes a caution against mistaking a white appearance of the tongue for an evidence of disease. Persons, he says, often on rising in the morning, look at their tongue, and finding it white, conclude that they are bilious and take physic. Such conclusions he assures us are generally erroneous; and the giving of a "*peristaltic persuader*" under such circumstances, he regards as being entirely unnecessary.

Lately we have examined the tongues of a number of persons in a state of health, and we found the appearances to be exceedingly diversified. In the case of a lady between 45 and 50 years of age, whose general health has always been remarkably good, and who was well at the time we made the examination, the tongue was as red as it usually is in acute gastritis, rather small, pointed, and very much fissured. In two other cases where the persons were in health the papillæ were very much enlarged, particularly at the upper ends of them, where they were of rather an umbelliferous form. In other instances the tongue was lobulated, sulcated, &c. Several cases we saw in which it was habitually furred without the conjunction of any inconvenience, other than that of a depraved taste of mornings.

It being now understood that serious disease may exist, of even the stomach itself, without being at all indicated by the tongue; and that morbid appearances of the tongue may coexist with a physiological exercise of the various functions, we will be better prepared to estimate the import of the different morbid appearances.

*What does a white appearance of the tongue indicate?* All know that in health the upper surface of the tongue is not of a bright red color. The tips of the villi being less injected with blood, than the lower parts, the tongue as a consequence, on its upper surface, is much paler than either the edges, tip, or under surface. This existing in conjunction with chlorosis might give rise to opinions that would be erroneous. Conjoined also with the exsanguineous condition common to some dyspeptics, or to females laboring under a nervous diathesis, it might be calculated to mislead; for in both cases it would be partly the result of a physiological cause. According to the time of day at which it is examined the tongue varies in appearance; its upper surface is much whiter early in the morning than after breakfast, at noon than after dinner, and at the end of a long fast than after the stomach has been fiilled. In part, this results from the motion to which the organ is subjected in mastication and deglution, by which a greater proportion of blood is invited into its substance; and also from the intimate sympathy there is between the tongue and stomach. A practice of taking cathartic medicines, epsom salts, aperient pills, and the like, obtains with some persons to an extent that creates a demand in the system for their habitual use. Of such persons the tongues, in nine cases out of ten, are coated white. Such an appearance is no evidence of disease, further than what might be supposed to result from the medicines acting on or irritating the mucous surface of the bowels.

After taking these things into consideration the white appearance of the tongue may be taken as an evidence that the system is some way or other out of order; and in connexion with other signs it will aid materially in making out the nature of the complaint. A very white appearance of the coat denotes that the disease is of recent origin, acute, and has rapidly invaded the system. This appearance is present in the commencement of acute inflammation, particularly inflammation of the bowels, in synochus fever of a high grade of arterial excitement; and in the epidemic erysipelas with which our country was recently visited; the coat in all the acute cases was at first of rather a snow-white appearance. A sallow white coat indicates a different grade of morbid excitement. It is the appearance most generally met with in typhus, dyspepsia, hypochondriasis, nervous diseases, chlorosis, and other diseases that consume considerable time in the period of incubation. Formed gradually, its color is modified to some extent by the low grade of arterial excitement in the tongue and by the vitiated secretions of the mouth.

There is another modification of the white coat which is generally regarded as a diagnostic of biliary derangement. I allude to the yellowish white found in jaundice, in what is generally called bilious fevers, and in yellow fever. When this appearance is present in conjunction with other symptoms, it may be of some importance in assisting to ferret out the condition of the liver; but in most of the cases that we witnessed of a late epidemic of bilious or malarial fever, the tongue exhibited no such phenomenon. The brown tongue is a modification of the white altered by the long continued presence of bilious matter, and by the depraved secretions of the mouth. Its presence, when connected with a tendency to dryness, as far as we have observed it, may be taken as an evidence that the disease, whatever be its character, has been of considerable duration, and is persistent in its course. Often the white coat accumulates to an extent that entitles it to the name of load. The import of a loaded tongue can seldom ever be misunderstood. To be formed it requires a considerable length of time, and of course, is evidence that the disease has been of considerable duration.

*What do the different colors of the tongue indicate?* Always it should be remembered, that the natural color of the tongue, as previously remarked, varies in different individuals. In those of a sanguine temperament, enjoying vigorous, robust health, it could not be expected to be of the same hue as in one of a lymphatic temperament, with a weak constitution and effeminate habits. In the former it would exhibit a very red healthy appearance; in the latter it would most likely be pale, blue, exsanguineous. Besides, any one who will take the trouble to examine a number of healthy tongues will find the shades of color as much diversified as are the complexions of the individuals examined. When not the result of the circumstances to which we have alluded, a red tongue may be regarded as a sign of disease general or local. In acute inflammation of the stomach or bowels, in high grades of febrile excitement, idiopathic or symptomatic, we generally have, in all parts of the tongue not covered with the coat, a very red appearance. As a consequence, this appearance, when coexisting with the original onset of the malady, or making its appearance during the course, is generally, and we believe in the majority of cases correctly, interpreted to signify, either primary acute disease of the stomach and bowels, or disease of some organ between which and the bowels there is a very intimate relation. A very red tongue, without being accompanied with any coat or load, would seem to augur the worst issue. Such however has not been verified by our experience. In all the instances of such a tongue, coming under our observation, the sequel of the complaint has been characterized by consequences of no worse character than attended a red tongue with coat or load. The tongue may be red with a clean smooth appearance, as though it were glazed, or it may be red and granulated. The former we have noticed as an original appearance in scarlatina and typhus, but it is oftener developed during the progress of the disease by some circumstance that removes the coat; the latter is sometimes present in chronic inflammation of the stomach, and we have witnessed it in some complaints in which the stomach was apparently not much in fault. It is probable that this granulated appearance is occasioned by the injection and hypertrophy of the papillæ on the edges and apex, the place where they are most generally observed. That both these appearances point to similar conditions of the bowels is a position that is generally proved by autopsical examinations. The red smooth tongue with a persisting tendency to dryness is always of serious import, and often coincides with a fatal issue. In relation to the granulations, Andral in his Medical Clinic, remarks, "they do not always remain in the same state: at times they are very prominent, very red and very numerous; at other times they are less apparent, paler, and fewer in number. Their development is always in the direct ratio of the intensity of the gastric irritation. Among the persons who present this particular state of the tongue, some have a stomach habitually sick; others have what is called a delicate or irritable stomach; they are forced to confine themselves to a certain regimen; they cannot without great inconvenience allow themselves the least excess in eating or drinking. This state of the tongue appeared to us more common in the gastric irritations of young persons than in those of persons more advanced in life. It is often observed in young females with a pale complexion and a weak constitution, whose stomach is habitually deranged; its existence may prevent one from considering and treating as nervous the symptoms they present on the part of the digestive organs. We have known several families, all the children of which presented this unusual development of the papillæ towards the apex of the tongue; in all there were at the same time other signs of gastric irritation. In one of these families the mother and her four daughters presented this particular appearance of the tongue, and all five had a very delicate stomach."

A blue or purplish state of the tongue is most likely caused by a feeble circulation. It is seldom, if ever, found in acute diseases where the circulation transcends the normal standard. In diseases of the heart with great lividity of the prolabium and countenance, the tongue and internal mouth partake of the general discoloration (Hall). The same is true of other complaints in which the nervous energy that keeps the heart performing its healthy function is interrupted or partially suspended, as for example in paralysis, several diseases of the nervous system, chlorosis, in purpura hemorrhagica, and in some forms of dyspepsia. This condition we have also witnessed in angina maligna, and in some cases of typhus. In the general, it should be regarded as indicating either primary disease of the heart, or an impaired state of the circulation from other causes. In angina maligna we have thought it due principally to defluxions in the vessels of the throat, which prevented the return of blood from the tongue to the heart.

Paleness of the tongue is usually, we believe, connected with lesions of the function of nutrition. Like some other of the phenomena, it is associated with a similar appearance of the general complexion. The tubercular disease when uncomplicated with gastric irritation—scrofula, chlorosis, and dyspepsia, resulting from atony of the stomach, together with several forms of hysteria, are most generally attended with a pale tongue, at times translucent at its edges, and occasionally swollen. When excessive, hemorrhage also gives rise to the same appearance. Paleness therefore may be regarded as reflecting among other things primary trouble in the assimilative functions, disordered hæmatosis, or waste of blood.

*What do the alterations in the form of the tongue indicate?* The tongue may be increased much beyond its natural size, or it may be diminished, lobulated, fissured, chapped, or indented. An enlarged or hypertrophied state may be connected with an exalted condition of the principal functions of life, or it may be associated with a condition exactly the reverse. In glossitis the tongue is often very much enlarged. The same is perhaps true in cases of variola and scarlatina. Enlarged from causes of this character it is always very red; and there are febrile excitement of a high grade, and other urgent symptoms present. Cachexia, from deranged digestion, disordered liver, or a dropsical diathesis, gives rise to a swollen pale state of the the tongue. In chlorosis this state is characteristic, and the most frequently developed. Here, besides being pale, it is indented, sometimes sulcated, with an almost constant tendency to tumefaction. As a general rule, there is no fever associated with this condition. It is, no doubt, caused by the presence of morbid fluids in its substance, something similar to the tumefaction that takes place in the face and extremities in certain morbid conditions of the system, and may be looked upon as an evidence that the complaint has been of great duration, for it requires considerable time for its formation. Indeed, the physician, on inspecting a tongue of this description, is often able to surprise his patient by the accuracy with which he can detail the history and symptoms pertaining to the case. A tongue less than the natural size we have so seldom witnessed that the class of diseases with which it is most constantly connected, as well as its indications, are, to us, matters more or less surrounded with doubt. About the same may be said of the lobulated modification of the form of the tongue; we have, however, never seen it except in persons advanced in life. It is caused perhaps partly by a shrinking of the substance of the muscles of the tongue, by which the surface is corrugated or formed into lobes resembling those of the cerebellum. An indented tongue always indicates great prostration and a want of cohesive force in the solids. It is found in the advanced stage of adynamic diseases, existing in conjunction with paleness and tumidity. What the import of a fissured tongue is, remains to be noticed. It is observed in sickness and in health, in the delicate and in the robust, in connexion with every variety of temperament, and in diseases entirely opposite in their nature. It is, however, like the lobulated appearance, peculiar to advanced life. The fissures may be few in number, and traversing the surface in a particular direction, or they may be very numerous, cutting it up into various forms. Existing in conjunction with an acute disease that produces a tendency to dryness, the fissured appearance would impart to the tongue a chapped appearance, that might be delusive, for it would import a high degree of gastric or intestinal irritation that perhaps would not be present.

Dryness of the tongue must always be regarded as a very grave phenomenon. Sometimes it is present at the commencement of a disease; oftener not until sometime afterwards. It is generally observed in connexion with a sthenic diathesis, inflammation, fever, local injuries, &c. It is liable to occur in inflammation of most of the organs and tissues, particularly in inflammation of the alimentary canal, whether primary or as the result of diseased action in other parts of the system. Usually the action of the heart and arteries is above the healthy standard. Perhaps this is always the case except where the dryness supervenes upon a system worn down by disease or just before *articulo mortis.* A dry state of the tongue is always associated with redness; sometimes the surface is clean, at other times loaded and chapped, once in a while black. The degree of dryness varies according to the stage of excitement. In fever it is always greatest during the exacerbation; and indeed it frequently happens that this is the only period in the complaint in which the tendency to dryness is at all observable.—Again, dryness is apt to be present in the advanced stage of most diseases that terminate fatally. Evidently dryness originates from a suspended state of the secretions proper to the tongue and inside of the mouth. In the majority of cases it is a measure of the deranged condition of the secretions generally; and, although not always the case, it is pretty strong evidence of gastro-intestinal irritation. Very true it is that we may have a tendency to dryness of the tongue from some affection of the organ itself, or from disease of the mucous membrane lining the mouth, without much constitutional disturbance. And it is well known that we may have lesions of a very severe character in the stomach or intestines without giving rise to any abnormal appearance at all in the tongue, much less to dryness. Still we regard these as being exceptions to a general rule, and as a consequence look upon dryness as being among the most serious appearances with which the tongue can be affected.

It remains for us to make some suggestions on the import of several of the more prominent changes which take place in these morbid appearances, and also to say something concerning depraved taste, and the function of taste.

Pertaining to the color, there are certain changes which, by the intelligent practitioner, are well appreciated. In cases where the tongue has been very pale, as for example, in chlorosis, and commences returning to something like the natural color, amendment may be pretty safely predicted. Such a change would indicate that a salutary impression had been made on the disease, and that the organism was returning to a physiological condition. Of the blue, exsanguineous, or limpid color common to some forms of heart disease, the same might be stated. The change would indicate a better state of the function of hematosis. A change from unnatural redness to the common appearance, is always regarded as ominous of favorable alterations in the character of the complaint, especially when seated in the stomach or bowels. Changes relating to the coat possess more or less interest. When this is noticed to be increasing in thickness and extent it may safely be affirmed, that the malady is in a state of progression. On the contrary, when it begins to peel off, leaving the surface beneath it of a normal appearance, the state of things, as a general rule, may be regarded as being favorable. To this, occasionally, we find some exceptions; sometimes, as in protracted fevers, it happens that a cleaning off takes place several times during the course without being attended with any visible improvement in the symptoms. In such, the surface beneath is found very red after the coat leaves it, and in many instances disposed to dryness and chaps. The color of the coat undergoes several changes which in this place might be noticed. It assumes in some complaints a dirty or brownish hue; in others it grows rather yellow. The former indicates tenacity and persistence in the course; the latter the approach or presence of biliary complication. A tongue that, during the whole course, continues to grow dry is, *cæteris paribus*, indicative of an unfavorable issue. On the contrary, where dryness has made its appearance early, and the organ afterwards exhibits a tendency to moisture the prospects of a recovery are very much increased. In many acute diseases we have the tendency to dryness for some considerable time existing in connexion with a similar state of the surface, palms of the hands, &c. While this continues the prognosis is equivocal. No sooner, however, does moisture make its appearance, than doubts are dissipated, and recovery may be predicted with some considerable degree of assurance.

The alterations which take place in the function of gustation afford in many instances evidence of a valuable character. Besides a perverted state of the function of the nerve of taste arising from the influence of the morbid cause, there are also other circumstances that exercise a prejudicial agency over the gustatory function. We allude to the coat on the tongue by which the sentient extremities of the nerve are so much covered up as either to prevent all contact with alimentary substances, or only such as gives rise to depraved taste. Subsidence, therefore, of depraved taste, and the return of the healthy action concerned in the function, may be regarded as ominous of amendment, and the return of the system to a healthy state. Some complaints we have witnessed in which the taste underwent but little if any change. But in such cases the coat has been either absent or very thin.

Related to the motions of the tongue there are some things that may at times be consulted with advantage. A tremulous state is frequently witnessed in typhus and its allies, and as a general rule, might be taken to import a debilitated condition of the system. Occurring in connexion with a nervous diathesis, or actual disease of the nervous system, its indications would be different. Partly it might then also be due to debility, but more likely to a lesion of innervation. As yet we have not witnessed a case of chorea or paralysis in which the tongue partook of the tremor incident to the part or parts affected. We can see, however, no good reason why this should not occasionally be the case. The want of power exhibited by some patients to protrude the organ over the teeth for inspection, no doubt, depends upon the same cause or causes giving rise to tremor, and should be regarded as being connected with about the same condition of the system.

June 25th, 1846.

